# Network Representation of fMRI Data Using Visibility Graphs: The Impact of Motion and Test-Retest Reliability

**DOI:** 10.1007/s12021-024-09652-y

**Published:** 2024-02-09

**Authors:** Govinda R. Poudel, Prabin Sharma, Valentina Lorenzetti, Nicholas Parsons, Ester Cerin

**Affiliations:** 1 Mary Mackillop Institute for Health Research, Australian Catholic University, 215 Spring Street, Melbourne, 3000 Australia; 2 Department of Computer Science, University of Massachusetts, Boston, MA USA; 3 Neuroscience of Addiction and Mental Health Program, The Healthy Brain and Mind Research Centre, School of Behavioural and Health Sciences, Faculty of Health Sciences, Australian Catholic University, Melbourne, Australia; 4 School of Psychological Sciences, Monash University, Melbourne, Australia; 5 Braincast Neurotechnologies, Melbourne, Australia

**Keywords:** Visibility graph, Resting-state fMRI, Brain network analysis, Timeseries features

## Abstract

Visibility graphs provide a novel approach for analysing time-series data. Graph theoretical analysis of visibility graphs can provide new features for data mining applications in fMRI. However, visibility graphs features have not been used widely in the field of neuroscience. This is likely due to a lack of understanding of their robustness in the presence of noise (e.g., motion) and their test-retest reliability. In this study, we investigated visibility graph properties of fMRI data in the human connectome project (*N* = 1010) and tested their sensitivity to motion and test-retest reliability. We also characterised the strength of connectivity obtained using degree synchrony of visibility graphs. We found that strong correlation (*r* > 0.5) between visibility graph properties, such as the number of communities and average degrees, and motion in the fMRI data. The test-retest reliability (Intraclass correlation coefficient (ICC)) of graph theoretical features was high for the average degrees (0.74, 95% CI = [0.73, 0.75]), and moderate for clustering coefficient (0.43, 95% CI = [0.41, 0.44]) and average path length (0.41, 95% CI = [0.38, 0.44]). Functional connectivity between brain regions was measured by correlating the visibility graph degrees. However, the strength of correlation was found to be moderate to low (*r* < 0.35). These findings suggest that even small movement in fMRI data can strongly influence robustness and reliability of visibility graph features, thus, requiring robust motion correction strategies prior to data analysis. Further studies are necessary for better understanding of the potential application of visibility graph features in fMRI.

**Supplementary Information:**

The online version contains supplementary material available at 10.1007/s12021-024-09652-y.

## Introduction

A time series can be mapped into a network by linking signal visibility at each timepoint with respect to other timepoints, an approach known as visibility graphs (VG) (Lacasa et al., 2008). A VG network captures intra-time-series relationships, which can be analysed using methods from graph theory. This analysis can generate rich set of features useful for characterising the structure and dynamics of the time-series data (Sannino et al., 2017; Varley & Sporns, 2022). One key advantage of VG analysis is its ability to handle nonlinear and nonstationary time-series data (Sannino et al., 2017; Varley & Sporns, 2022). Studies have shown that VG inherits properties of the corresponding time-series into its topological structure. VG properties of periodic time-series are also useful for capturing information hidden in multivariate, non-stationary, and noisy datasets (Sannino et al., 2017; Stephen et al., 2015), which can be employed for event detection, classification, temporal community detection, and data mining applications in neuronal time-series data (Supriya et al., 2021; Tang et al., 2013; Zhang et al., 2022; Zhu et al., 2014a).

The use of VG-based analysis of neuronal signal data as networks is still in its infancy, with most of studies focusing on univariate analysis of EEG time series. One of the first applications of VG analysis to EEG data was for classifying sleep stages using a single channel EEG signal (Zhu et al., 2014a), whereby topological features of epoch-by-epoch VG networks were employed for the classification of EEG segments into different sleep stages. Network features, such as edge strength, have also been found to be useful for classifying sleep states (Supriya et al., 2021). Furthermore, VG analysis can detect abnormal neuronal events (e.g., seizures) from EEG data. For example, a VG-based approach was found to be more sensitive to seizure detection than a simple entropy method (Tang et al., 2013). Other studies have found that the graph theoretical features of VG to be highly effective in discriminating epileptic seizures from EEG time-series data (Supriya et al., 2016; Zhu et al., 2014b).

Few studies have used a VG-based approach for the analysis of fMRI data (Gao et al., 2020; Varley & Sporns, 2022). fMRI captures the rich spatiotemporal dynamics of human brain activity, which can be used to characterise brain network correlations, temporal evolutions, and state transitions associated with human behaviour. The spatiotemporal nature of fMRI makes it amenable to multilayer transformation, enabling singular representations of both intra and inter-regional correlations (Varley & Sporns, 2022). A recent proof-of-concept study used this multilayer VG approach to analyse correlations between brain regions and, for the first time, demonstrated that this approach may be used to classify different disease states (Sannino et al., 2017). Another study (Gao et al., 2020) used graph theoretical features from VG to distinguish individuals with Alzheimer’s disease from healthy controls. Although these findings are very encouraging, there is a need for a better understanding of the robustness and reliability of VG features in resting-state fMRI data. Furthermore, the characterisation of the different network properties describing the structure of VGs within neuronal processes as encoded in fMRI time-series is missing in the literature.

The most common approach for fMRI-based brain network analyses utilizes direct comparison of time-series using similarity metrics such as the Pearson correlation coefficient. Visualization Graphs (VGs) provide a unique framework that goes beyond mere pairwise correlations, allowing for the extraction of complex relationships within the brain’s functional architecture in both time and space. The construction of VGs involves mapping the time series data to a graph structure based on the visibility algorithm, capturing dependencies in time that may be overlooked by methods solely relying on pairwise correlations (Stephen et al., 2015; Zheng et al., 2021). Furthermore, VGs enable the estimation of associations across time and extraction of multivariate graph theoretical features from univariate time series. VGs also enable the exploration of higher-order associations and dynamics within the brain’s functional network, providing a more nuanced understanding of the complex relationships present in fMRI data.

Despite the increasing use of VG analysis in network neuroscience studies utilising fMRI data, there is a lack of research on the robustness and reliability of measures generated through this analysis. fMRI signal is highly sensitive to any movement inside the scanner, which can lead to transient and slow signal changes (Power et al., 2015). The presence of motion artifacts in signal data poses a significant challenge to VG (Variance-based Global) analysis and its applications. Both the transient and slower and more widespread (global) signal changes introduced by motion artifacts may obscure meaningful variations in the data. This can significantly affect the graph structure of VGs, leading to the formation of additional edges or the suppression of existing edges (Ahmadlou & Adeli, 2012; Donner & Donges, 2012; Varley & Sporns, 2022). Furthermore, test-retest reliability of fMRI activity over multiple sessions/days is poor (Noble et al., 2019), which may impact on the reliability of associated VG graphs. This study aimed to evaluate the impact of varying levels of motion on the graph theoretical properties of VGs, and to characterize the reproducibility of these measurements when the same individuals are tested multiple times (test-retest reliability).

## Methods

### Mapping Time Series to Networks Using Visibility Graph

A time series can be converted into a network by mapping the connection between timepoints using visibility criteria (Lacasa et al., 2008; Zhang et al., 2022). A visibility graph (VG) for a time series *x*_*i*_ is defined as a graph G = (*V, E*) such that each time stamp, *t*, is a node (*V*) in the graph and the edge (*E*) between nodes 𝑣_𝑖_, and 𝑣_𝑗_, is a line of visibility between the signal amplitudes *x*_*i*_ and *x*_*j*_.

Any two data points (t_i_, x_i_) and (t_j_, x_j_) will be connected nodes of the graph if any other data points (*t*_*k*_, *x*_*k*_) which lie between them meet the following criteria.

$${x}_{k}< {x}_{j}+ {(x}_{i}-{x}_{j}) \frac{({t}_{j}-{t}_{k}) }{({t}_{j}-{t}_{i}) }$$

**Fig. 1:**
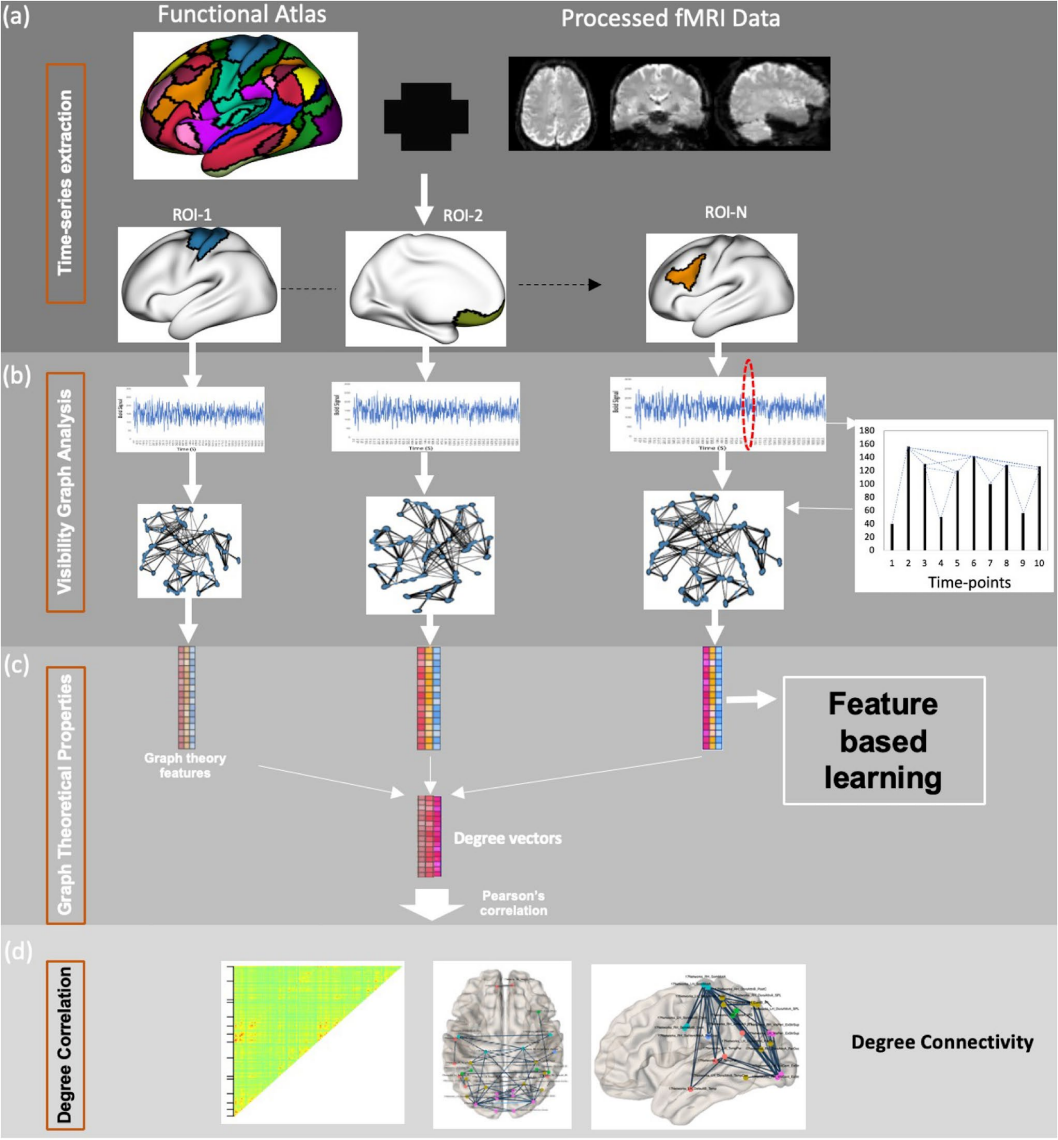
A framework for visibility graph analysis of fMRI data. **a** fMRI data is parcellated into different brain regions using an atlas **b** Average time series is extracted from all brain regions. This time-series data can be represented as a temporal landscape such that visibility between each data point can be identified. Visibility between two time points exists (i.e., an edge) if any other time point between them has a corresponding intensity below the line connecting the two time points (i.e., there is a direct line-of-sight between the peaks of time points) **c** The graphs generated using the visibility criteria has number of nodes equal to number of time points in the data. The graphs can then be processed using standard graph theoretical analysis methods to generate graph features. **d** The visibility graph degree vectors from each ROI can be correlated to generate a Degree Connectivity network, providing a new measure of functional connectivity

This natural visibility can also be illustrated by using vertical bars to represent time-series data (Fig. 1b). These VGs are connected graphs, in which each node has at least one connection (i.e., neighbouring timepoints are connected to each other). This network is invariant under affine transformations and vector translations of the data. Weighted VGs (Silva et al., 2022) can be obtained by using Euclidian distance between timepoints as weighting factor such that:

$${w}_{(i,j)}= \frac{1}{\sqrt{{{(t}_{j }- {t}_{i}) }^{2}+{{(x}_{j }- {x}_{i}) }^{2} }}$$

VG graph of fMRI time-series represent a simplified mathematical construct to generate graph format representation of timeseries. Each node in the graph represents a time-point and the binary edges represent visibility of signal at a timepoint to another. Thus, edges represent temporal relationship in the data. VG networks are useful for generating unique features, which can reveal certain patterns or relationships within the time series data. However, it cannot provide insights into the specific neural connections, synapses, or the underlying spatial architecture of the brain (Silva et al., 2022).

An important aspect of VG analysis is that there is a natural correspondence of nodes (i.e., timepoints in VG) across different brain regions and subjects. Hence, any local features of nodes such as degree-sequence can be compared across various brain regions. One previous work has used this natural alignment within multiplex visibility framework as compact way of extracting at once both the local temporal structure and the global connectivity pattern (Sannino et al., 2017). Our current work aimed to go back one step and investigate whether basic local and global properties of visibility graphs are reliable and robust.

### Graph Theoretical Features of Visibility Graphs

The variability of time-series data can be analysed using graph-theoretical methods. These analyses can extract features such as centrality, distance, community structure, and connectivity, which are important for understanding the characteristics of a graph. In this work, we use five global properties of the graph - average weighted degree, average path length, global clustering coefficient, number of communities, and modularity - to characterise the time-series graphs. A brief description of these measures is provided below.

The average weighted degree is the arithmetic mean of the weighted degrees of all nodes in a network. The weighted degree is a measure of the strength of connectivity for each node and characterises the intensity of connectivity in the node’s neighbourhood. The average path length is the mean of the shortest paths between all pairs of nodes. This is a measure of the flow of information in the network. The global clustering coefficient is a measure of the extent to which the nodes of a graph tend to cluster. It measures the probability that two nodes connected to a given node are also connected. The number of communities is the number of clustered groups of nodes in the network. The modularity, Q, measures how well the graph can be divided into communities. A high modularity indicates a graph with a dense internal community structure and sparse connections between nodes of different communities.

The Walktrap Community-finding algorithm was utilized for community detection. This algorithm employs a random walks approach to identify regions of the network where nodes are more likely to be interconnected, indicating the presence of a community. Furthermore, to calculate the modularity of a graph, we determined the degree of separation of nodes belonging to different communities using the approach described in a previous work (Silva et al., 2022). We used the NetF toolbox to generate VG features from fMRI timeseries (Silva et al., 2022). The toolbox is available via github (https://github.com/vanessa-silva/NetF).

### Network Connectivity Using Degree Synchrony

The VG networks can also be represented as a system of multilayer network in which each brain region forms a layer (Fig. 1) (Ahmadlou & Adeli, 2012). Such a multilayer network has one-to-one correspondence between nodes of each layer such that node *i* in one layer corresponds to the same node in other layers, making the multilayer graph a multiplex network. Graph theoretical features of multiplex graph can be used to measure similarity between layers.

The *degree synchronization* is a measure of similarity between the series of connectivity degree of each layer (Ahmadlou & Adeli, 2012). Since each node in each layer represent a time-point, the series of connectivity degree of visibility graphs at layers is a representation of fluctuations in degree over time. Thus, any correlations in fluctuations in visibility graph degrees over time across multiple layers represents an alternative measure of synchronization. Thus, degree synchronization is given as:

$$S \left[Dx, Dy\right]= \frac{\sum (Dx-\overline{Dx}) \left(Dy-\overline{Dy} \right)}{\sigma \left(Dx\right)\sigma \left(Dy\right)}$$

where Dx and Dy are degree sequence of visibility graphs representing timeseries x and y.

While correlation-based connectivity mapping of fMRI time-series provides valuable static insights into functional connectivity, degree synchrony-based connectivity mapping introduces a unique dimension, emphasizing how fluctuations in the amplitude of time signals synchronize across different regions over time. Although more work is needed to better understand the biological underpinnings of degree-synchrony-based connectivity mapping, it represents an important step towards a computationally efficient method with the potential to capture multi-scale dynamics in fMRI data.

### Application of Visibility Graph Analysis in rs-fMRI Data

#### Participants and Data

The dataset used in the present study was obtained from the Human Connectome Project (HCP) S1200 release (Van Essen et al., 2013). Participants (*n* = 1113) were young healthy adults aged 22 to 37 years. Each participant took part in two sessions (conducted on two consecutive days) of resting-state fMRI scans acquired over two runs (right-to-left and left-to-right phase encoding) of 14m33s each. The MRI data were acquired on a customized Siemens Skyra 3 Tesla MR scanner using a multiband echo planar imaging sequence (TR = 720ms, TE = 33.1ms, voxel dimension: 2 × 2 × 2 mm^3^) (Smith et al., 2013). The current study uses data from the participants who completed all four runs with a final sample of 1010 individuals (mean age 29 ± 4 years, 453 males).

#### fMRI Pre-processing

We used fMRI data processed using HCP pipeline and available publicly. The detailed pre-processing steps used in HCP pipeline are described elsewhere (Glasser et al., 2016; Smith et al., 2013). Briefly, the fMRI pre-processing steps within the HCP data included (1) removal of spatial artifacts and distortions, (2) correction of head motion, (3) spatial registration to the MNI (Montreal Neurological Institute) standard space and (4) removal of motion-related and structured physiological noise artefacts using ICA-FIX (Salimi-Khorshidi et al., 2014). Data were analysed in CIFTI (Connectivity Informatics Technology Initiative) format, in which cortical surface time series and subcortical volume time series were structured in a grayordinate dense time series file. Head motion was quantified using framewise displacement (Power et al., 2015).

#### Estimation of Visibility Graph Features

The pre-processed fMRI time-series data from each session (REST1 and REST2) were temporally concatenated across the two runs within the session. This generated approximately 30 min of rs-fMRI data per session. The fMRI data were parcellated using Yeo-17 functional atlas (Yeo et al., 2011) for obtaining average time series across functionally-defined brain regions (114 brain regions). The time-series data obtained from the parcellation were then converted into VGs using natural visibility graph algorithms in R (Silva et al., 2022). To characterise the degree properties of the VG obtained from fMRI time-series, log-log degree distribution was estimated for each subject. The degree distributions were obtained from several regions of interests within the Yeo-17 atlas for demonstration purpose. The degree distribution plots were pooled across participants and plotted. Power-law fit was obtained by using powerLaw package in R. The VG for each region was analysed using graph theoretical analysis tools in implemented in R by a previous work (Silva et al., 2022) to obtain the five features: average weighted degrees, average path length, global clustering coefficient, number of communities and modularity. In order to characterise degree distribution of VG associated with fMRI time-series, log-log plot of degree distribution were generated for a region from each canonical functional network. Power law fit was approximated for each degree distribution plot using PoweRlaw package in R.

### Impact of Motion and Test-Retest Reliability

To identify the impact of motion intrusions on VG features and identify the threshold required for removing any motion related impacts, we characterised the relationship between proportions of motion corrupted data and VG features across the participants. First, to estimate the impact of motion on the features, we calculated the correlation (Pearson’s) between each feature and the percentage of fMRI data points associated with motion across the participants. This percentage was defined as the percentage of fMRI data points associated with frame-wise displacement (FD) greater than 0.2 mm (i.e., data points with greater than 0.2 mm FD divided by total data points). Next, to determine the allowable percentage of data points with motion without compromising the VG features, we ran the correlation analysis using various subsets of the data, with each subset exclusively comprising subjects exhibiting less than x% of frames characterized by frame displacement (FD) exceeding 0.2 mm. The value of x ranged from 10 to 40% in the steps of 0.5%. This allowed us to identify appropriate motion thresholds (in terms of tolerable percentage of motion corrupted data points) necessary for reliable VG features.

To estimate test-retest reliability of VG features between two sessions acquired on different days (REST1 and REST2), we computed the intraclass correlation coefficient (ICC). A two-way mixed effects model with absolute agreement as a reliability measure was used, as per the recommendation from previous work (Koo & Li, 2016). This analysis was applied only to the low motion dataset identified using the motion threshold established in the previous section.

### Functional Network Estimation Using Degree Synchrony

Mapping of functional network using VG degree synchrony is a novel approach. Only one previous study has attempted to do this in a small dataset (Gao et al., 2020). We used the low motion fMRI dataset and mapped the functional networks in the brain using the degree synchrony. We generated degree synchrony based functional connectivity maps for all individuals, which were averaged to obtain a mean connectivity map.

The primary motivation for this analysis was to introduce methodological approach for generating these networks, paving the way for future in-depth analyses. This analysis should be taken as an initial step in hypothesis generation, opening the door for subsequent detailed analyses. The detailed analysis of network properties and the impact of motion on these analyses is outside the scope of this manuscript.

To capture the degree synchrony, a pairwise correlation analysis was performed on the VG degree time series of each region, yielding a matrix of inter-regional correlations. The construction of the functional network was achieved by thresholding the degree synchrony matrix to retain connections with significant synchrony.

## Results

### Degree Characteristics of fMRI Visibility Graphs

**Fig. 2:**
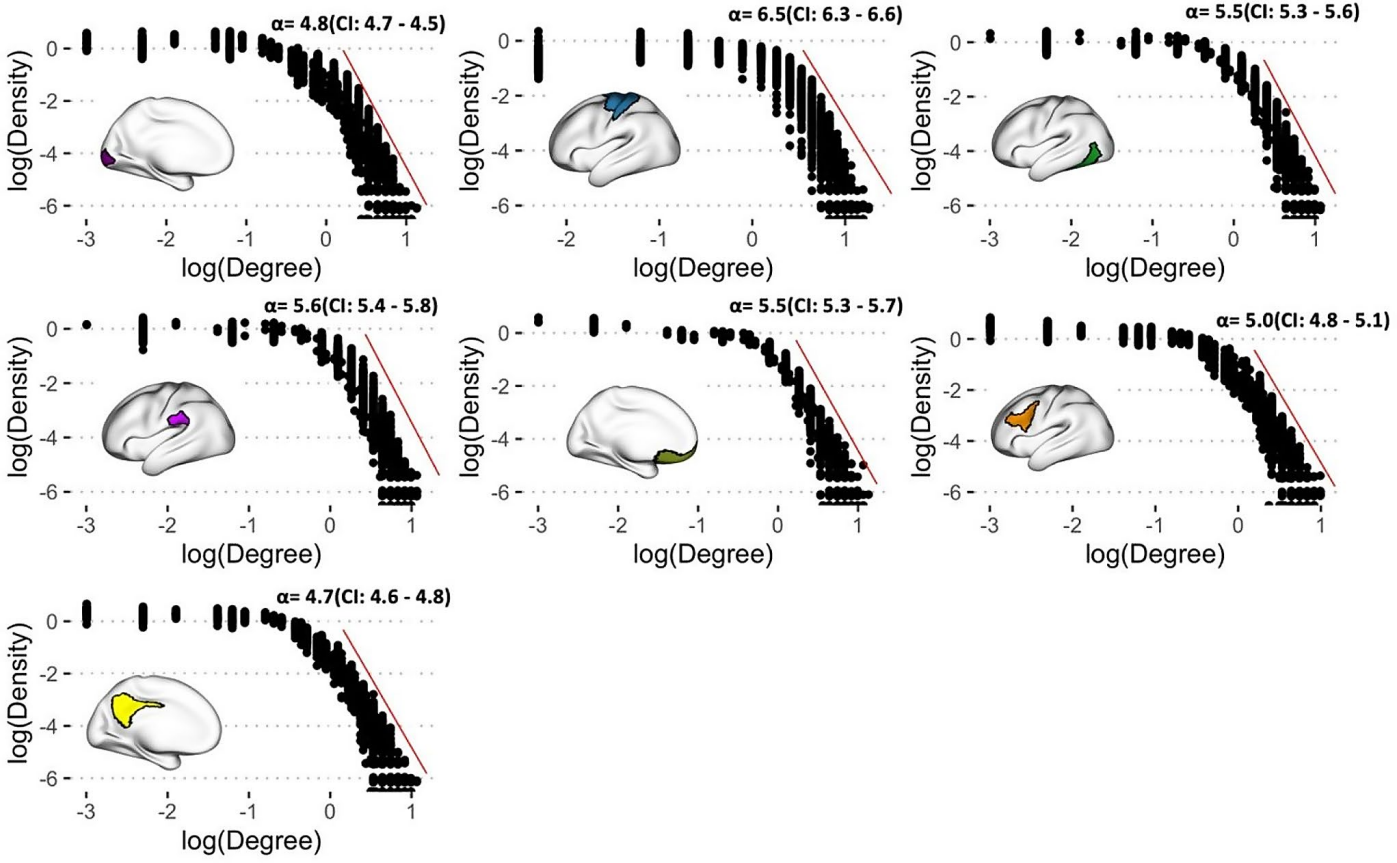
Log-log plot of weighted degree distribution of fMRI VG graphs. Each panel shows pooled degree distribution of average fMRI time series obtained from 7 example brain regions belonging to the resting-state networks. Alpha values (and 95% CI) of power laws fit for each region is also provided on the panels. Alpha values of power law fits were obtained for pooled distribution and averaged across participants

The log-log plot of degree distribution of fMRI weighted VG graphs in the example brain regions belonging to the seven resting state networks within the Yeo-17 atlas is shown in Fig. 2. The tail end of the distributions follows a power law distribution of the shape *P*(*k*) ∼ *k*^−α^, with the value of exponent alpha > 0 in all time-series across the canonical brain networks.

### The Impact of Motion on Visibility Graph Features

**Fig. 3:**
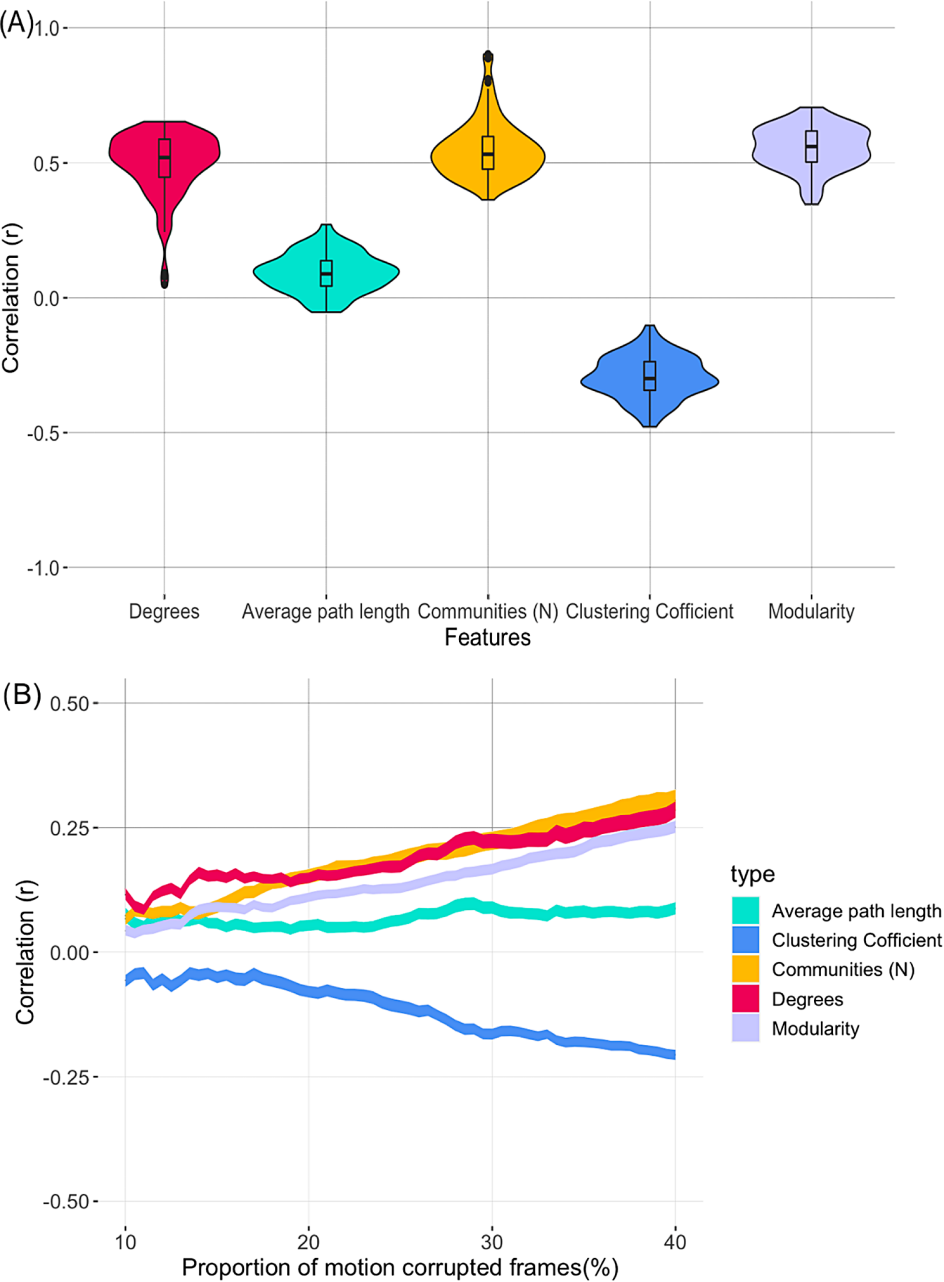
Association between percentage of motion corrupted data points and VG features across all the participants. **A** Violin plots showing summary statistics and density of correlation values between percentage of motion corrupted data points and VG features. The lower and upper hinges of boxplots within the violin plots correspond to the first and third quartiles (the 25th and 75th percentiles) of correlation values across the 114 brain regions. The density plots correspond to distributions of correlation values for the 114 brain regions **B** Changes in correlation between the percentage of fMRI data points associated with frame-wise displacement (FD) greater than 0.2 mm and VG features. Correlations are presented for different levels of motion in the data: from 10–40% of data corrupted by motion

Figure 3A shows the relationships between percentage of motion corrupted data points and VG features across all the participants. The largest impact of motion was observed on the number of communities (*r* = 0.55 ± 0.11), modularity (*r =* 0.55 ± 0.08), and average degrees (*r* = 0.50± 0.12), followed by the clustering coefficient (*r* = -0.29 ± 0.08) VG features. The average path length feature was least affected by motion (*r* = 0.09± 0.07). The relationships between the varying percentage of motion corrupted data points and VG features (Fig. 3B) shows that the impact of motion on VG features reduces more-or-less linearly with the reduction in the percentage of motion corrupted data points. On average, the correlation between motion and VG features falls to less than *r* = 0.1 when the motion corrupted data frames are less than 20%. A similar pattern of the impact of motion was observed in the second session (‘Rest2’) fMRI data (see Supplementary Figure S1).

### Test-Retest Reliability of the Visibility Graph Features

**Fig. 4:**
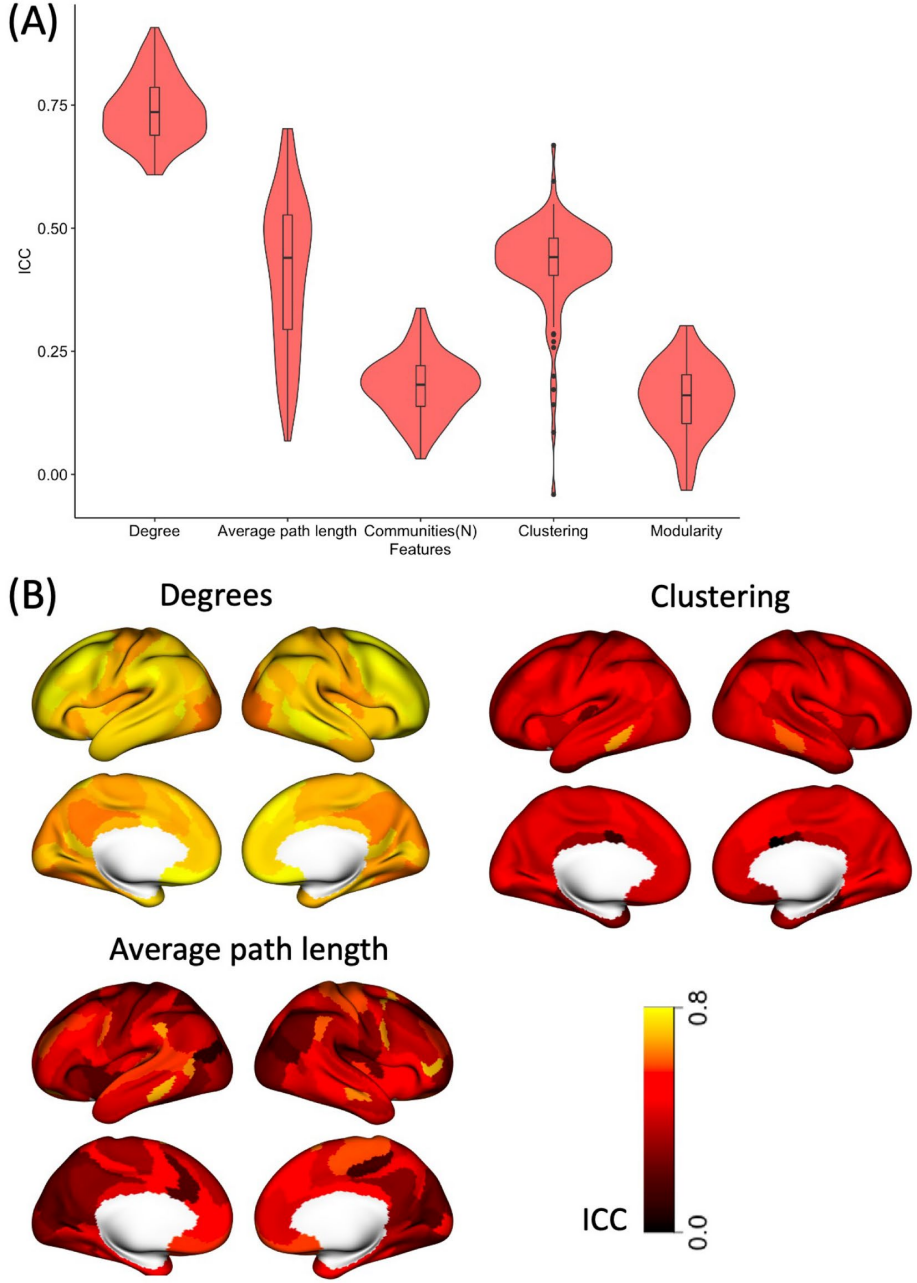
Test-retest reliability of VG features. **A** Violin plots showing summary statistics and density of intraclass correlation (ICC) grouped by VG features. The lower and upper hinges of boxplots within the violin plots correspond to the first and third quartiles (the 25th and 75th percentiles) of correlation values across the 114 brain regions. The density plots correspond to distributions of correlation values for the 114 brain regions **B** Spatial distribution of ICC values across the brain for degrees, clustering coefficient, and average path length VG features. The colour-bar represents ICC values

Due to the strong impact of motion on VG features, we used a subset of the datasets (*n* = 396) in which the percentage of motion corrupted fMRI data points (i.e., FD > 0.2 mm) was less than 20%. The intraclass correlation analysis within this dataset showed a high test-retest reliability between two sessions (Rest1 and Rest2) for the average degrees VG feature (ICC = 0.74, 95% CI = [0.73, 0.75]). The clustering coefficient (ICC = 0.43, 95% CI = [0.41, 0.44]) and average path length (ICC = 0.41, 95% CI = [0.38, 0.44]) also showed moderate reliability (Fig. 4A). Whereas the number of communities (ICC = 0.18, 95% CI = [0.17, 0.19]) and modularity (ICC = 0.15, 95% CI = [0.14, 0.17]) had low test-retest reliability. Figure 4B shows spatial variability in ICC values for degrees, average path length, and clustering coefficient features, which had moderate to high reliability. The reliability of the average degree feature was high across the brain (ICC > 0.5). For the clustering coefficient, 17 brain regions showed high reliability (ICC > 0.5). Whereas for the average path length, 38 brain regions showed high reliability.

### Degree Synchrony Based Functional Connectivity Maps

**Fig. 5:**
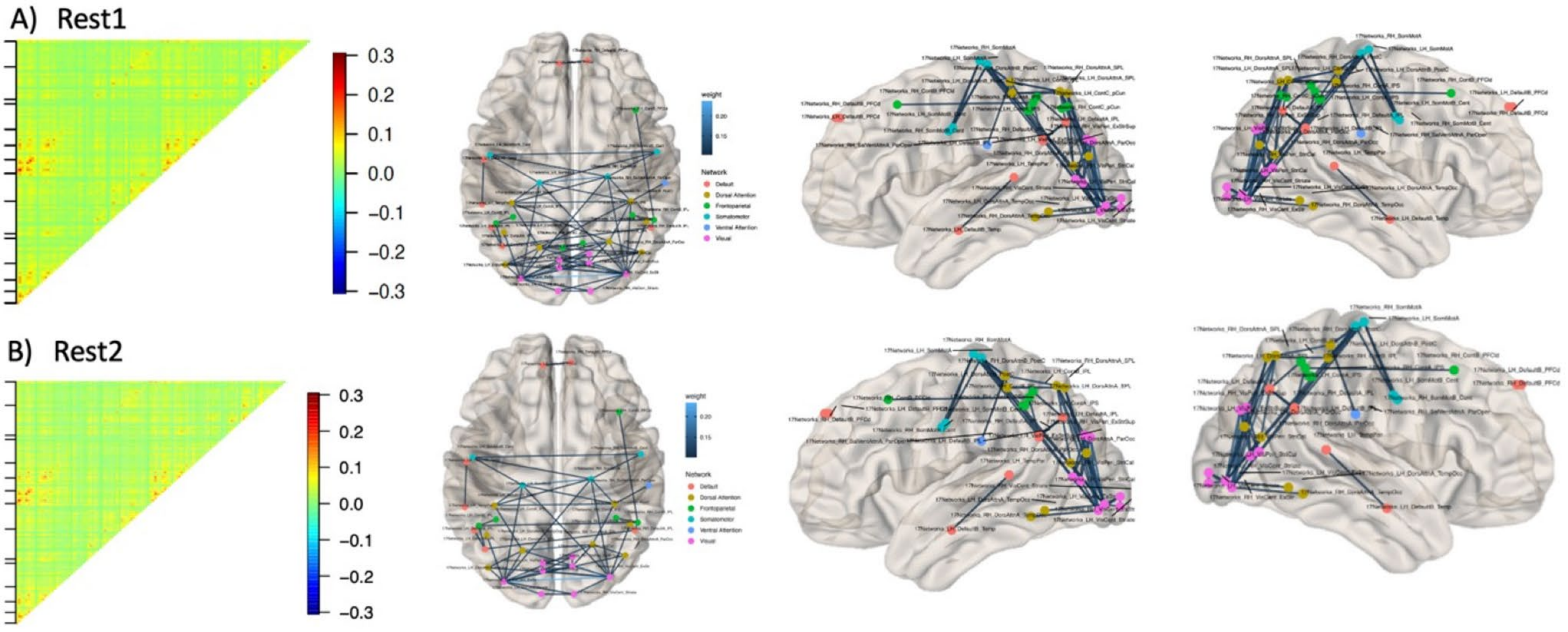
Functional connectivity maps obtained using degree synchrony measure from **A** Rest1 and **B** Rest2 sessions. The maps were highly consistent between session. The connectivity matrix represents upper triangle of the connectivity matrix, showing correlation between 114 cortical brain regions within the Yeo-17 atlas

Figure 5 shows the connectivity maps obtained by correlating degree vectors from visibility graph obtained from 114 cortical brain regions within the Yeo-17 atlas. The average connectivity values were low to moderate (< 0.4) in both sessions. In both sessions, most strongly correlated brain regions were bilateral brain regions within the salient and visual networks.

## Discussion

There is currently a lack of research on the reproducibility of the VG properties of fMRI data, which is a significant concern given the increasing use of VG analysis in network science studies involving fMRI data. This study aims to address this gap by evaluating the VG properties of fMRI time-series data from the Human Connectome Project (Van Essen et al., 2013). Specifically, the study aims to characterise the impact of motion on the VG properties of fMRI data, as well as the test-retest reliability of these properties between fMRI sessions. The findings show that the degree distribution of the VG associated with fMRI time-series follows a power-law distribution, suggesting scale-free nature of the VG networks. Furthermore, small movements can significantly alter the VG network structure and associated features. When a low motion is used the analysis, network features such as the average degree, average path length, and clustering coefficient show moderate to high test-retest reliability between sessions.

The presence of motion in fMRI data is problematic for interpreting both time-series and connectivity analyses (Poskanzer et al., 2022; Power et al., 2015). Motion can introduce noise and artifacts into the data, making it difficult to accurately interpret the connectivity between brain regions. Even small amounts of motion (FD ~ 0.2 mm), such as those caused by sleepiness in the MRI scanner (Poudel et al., 2021), can affect the VG features of fMRI time-series data. In this study of healthy control subjects, we found a strong correlation between region-wise VG features and the amount of motion-related intrusions in the data. We also found that VG features were most sensitive to motion when more than 20% of the data was contaminated by framewise displacement of more than 0.2. This level of sensitivity to motion is consistent with previous research showing a strong association between functional network features and framewise motion in fMRI data (Raval et al., 2022). VGs are particularly sensitive to the presence of outliers and large values in the data, which is typical of what happens when there are motion events in fMRI. Outliers can significantly affect the graph structure of VGs, leading to the formation of additional edges or the suppression of existing edges (Ahmadlou & Adeli, 2012; Donner & Donges, 2012; Varley & Sporns, 2022). This can make it more difficult to accurately interpret the properties of the VG, such as its degree distribution or clustering coefficient. Similarly, large values in the data can also impact the structure of VGs, causing the formation of additional edges or the suppression of existing edges. It is, therefore, important to carefully consider the impact of outliers and large values when using VGs for data analysis.

We also evaluated the reliability of the graph metrics of VG graphs using a low motion subset of the dataset. Graph metrics were compared using ICC statistics, which identified absolute agreement between two measurements and provided a measure of reproducibility (Koo & Li, 2016). The findings suggest that the reliability of VG metrics ranges from moderate to high, and is highly dependent on the type of metrics and brain regions. The average degree was observed to have the highest reliability amongst the graph measures used. Other measures, including the clustering coefficient and average path, also showed moderate reliability. Although this is the first study to investigate test-retest reliability of VG features in fMRI data, previous studies have reported similar ICC values for the reliability of graph metrics functional connectivity data (Braun et al., 2012; Termenon et al., 2016).

Furthermore, we generated functional connectivity maps by correlating the degree vectors obtained from the VG representation of the low-motion fMRI timeseries data. The correlation values in these maps were low to moderate, suggesting that degree fluctuations are not tightly synchronised. While one previous study has used this approach to generate functional connectivity maps, the correlation values were not reported (Sannino et al., 2017). This notwithstanding, the functional significance of the degree-synchrony-based functional connectivity maps lies in its ability to reveal meaningful relationships and associations between different brain regions. An important observation is that the degree-synchrony based connectivity maps are localised in the default-mode and somatosensory networks. Of particular interest are the somatosensory networks, known to be strongly influenced by transient changes in brain states such as fluctuations in wakefulness and slow-eye-closures associated with microsleeps (Wang et al., 2016). It is possible that the connectivity maps capture these transient changes, as degree sequences are influenced by high-amplitude signal changes (leading to high visibility of that time point and hence increased degree). However, future investigations are necessary to understand the relationship between these observed correlations and potential changes in behaviour. The utility of this approach in obtaining coordinated activity patterns among neural regions and obtain novel insights into the functional organization of the brain needs to be further investigated.

Some comments regarding the pros and cons of VG-based methods for fMRI analysis are warranted. One of the main advantages is the simplicity and low computational complexity of the algorithm. This makes VG analysis particularly suitable for the spatio-temporally dense fMRI data. Additionally, VG-based methods can potentially uncover hidden structural properties of time-series, which may not be possible with conventional time-series analysis techniques (Donner & Donges, 2012). This is an active area of research and requires further work. The features generated from VG analysis of fMRI has potential use in data mining for fMRI-based classification and prediction of neurological conditions (Gao et al., 2020; Sannino et al., 2017). Despite the benefits, it is important to acknowledge the limitations that exist. For example, the neurophysiological basis for the network structure that is obtained from VG is not clear. In a biological context, a visibility graph obtained from a single brain region signifies the influence or interaction between neural activity at timepoints represented by the nodes. The edges in the VG network signify temporal relationships in the time series. For example, if node A and node B are connected by an edge, it implies that the neuronal activity at timepoint A is influencing or affecting the neuronal activity at timepoint B (or vice versa). Given the temporal order of neural activity, this concept is consistent with neuronal activity that unfolds over time, leading to a particular behaviour and the principle of predictive coding of brain function. Recent studies have shown that the ability of a neuron to influence its future activity may provide a mechanism for learning in the brain (Luczak et al., 2022). Furthermore, brain connectivity (between brain regions) obtained from VG features (e.g., degree connectivity) also provides a novel way of looking at how different neuronal populations might be interacting in the brain over time. Co-fluctuations in VG degrees (e.g., degree synchrony) likely represent temporal synchrony between neuronal populations that are largely driven by transient changes. Another challenge in interpreting the VG based measures is the lack of correspondence across individuals in the order of neural activity over time. Resting-state activity is characterized by a succession of intricate cognitive, emotional, perceptual, and motor processes that unfold uniquely in each individual scan (Gonzalez-Castillo et al., 2021). Hence, the fluid and evolving series of neural activity may not be directly comparable across individuals.

Another limitation is that the utility of the VG features and VG-based connectivity maps largely depends on their ability to reflect and predict human behaviour and neurological states. While the scope of the current study was limited to characterising the robustness and reliability of the VG features, it is important to also examine how these features are associated with age, sex, and different cognitive profiles. Furthermore, in this study, we used a low-motion dataset to generate degree-synchrony-based maps, thereby limiting the ability to investigate the robustness of degree-synchrony maps. Furthermore, computational resources required for the analysis of fMRI time series using VG scale exponentially when using a greater number of time points and a finer parcellation of brain regions (e.g., voxel-level analysis). This underscores the need for more research on robust and optimized algorithms and scalable solutions for running high spatiotemporal analysis using VG. Efforts to address and manage these challenges will be pivotal in ensuring the success of the VG approach in fMRI analysis. Other metrics (e.g., edge overlap, mutual information, transfer entropy etc.) that emphasise different aspects of connectivity dynamics could also be used to generate connectivity between brain regions. Future studies should explore these approaches to measure similarities in multilayer VG framework.

Overall, the results presented in this paper raises important questions about robustness and reliability of VG analysis of fMRI data. The high sensitivity of VG properties to motion is a particularly concerning issue and requires careful pre-processing of the data. While some VG metrics show good test-retest reliability, the need for stringent motion thresholds may limit the use of VG in fMRI data. In order to address these limitations, future research should investigate whether motion correction approaches can effectively overcome the issues identified in this work.

### Electronic Supplementary Material

Below is the link to the electronic supplementary material.

Supplementary Material 1

## Data Availability

Data were provided by the Human Connectome Project, WU-Minn Consortium (Principal Investigators: David Van Essen and Kamil Ugurbil; 1U54MH091657) funded by the 16 NIH Institutes and Centers that support the NIH Blueprint for Neuroscience Research; and by the McDonnell Center for Systems Neuroscience at Washington University. The R script associated with this study has been shared via github at (https://github.com/govin2000/vganalysis).
