# Supplementary material for: Network Representation of fMRI Data Using Visibility Graphs: The Impact of Motion and Test-Retest Reliability
Source: Neuroinformatics. 2024 Feb 9;22(2):107–18. doi: 10.1007/s12021-024-09652-y (PMC11021232; doi:10.1007/s12021-024-09652-y)
Supplement: Supplementary file 1 — Supplementary Material 1 [file 12021_2024_9652_MOESM1_ESM.docx]

**Figure S1:** Association between motion and visibility graph features in the resting state data from Session 2. (A) Violin plots of Pearson’s correlation between average framewise displacement and visibility graph features in 114 cortical regions as per Yeo-17 parcellation. The boxplot represent mean and 95% CI of correlation values across the brain regions. (B) Changes in correlation between percentage of motion frames in the data (i.e., >0.2 FD) and VG features. Correlation are presented for different levels of motion in the data, increase from 10% of data corrupted by motion to 40% of the data corrupted by motion.
